# Serine Phosphorylation of L-Selectin Regulates ERM Binding, Clustering, and Monocyte Protrusion in Transendothelial Migration

**DOI:** 10.3389/fimmu.2019.02227

**Published:** 2019-09-25

**Authors:** Abigail Newe, Karolina Rzeniewicz, Melanie König, Carsten F. E. Schroer, Justin Joachim, Angela Rey-Gallardo, Siewert J. Marrink, Jürgen Deka, Maddy Parsons, Aleksandar Ivetic

**Affiliations:** ^1^BHF Centre of Research Excellence, James Black Centre, King's College London, London, United Kingdom; ^2^Groningen Biomolecular Sciences and Biotechnology Institute, Groningen, Netherlands; ^3^European Molecular Biology Laboratory, Heidelberg, Germany; ^4^Randall Centre for Cell and Molecular Biophysics, King's College London, London, United Kingdom

**Keywords:** förster resonance energy transfer (FRET), fluorescence lifetime imaging microscopy (FLIM), molecular dynamics, extravasation, diapedesis

## Abstract

The migration of circulating leukocytes toward damaged tissue is absolutely fundamental to the inflammatory response, and transendothelial migration (TEM) describes the first cellular barrier that is breached in this process. Human CD14^+^ inflammatory monocytes express L-selectin, bestowing a non-canonical role in invasion during TEM. *In vivo* evidence supports a role for L-selectin in regulating TEM and chemotaxis, but the intracellular mechanism is poorly understood. The ezrin-radixin-moesin (ERM) proteins anchor transmembrane proteins to the cortical actin-based cytoskeleton and additionally act as signaling adaptors. During TEM, the L-selectin tail within transmigrating pseudopods interacts first with ezrin to transduce signals for protrusion, followed by moesin to drive ectodomain shedding of L-selectin to limit protrusion. Collectively, interaction of L-selectin with ezrin and moesin fine-tunes monocyte protrusive behavior in TEM. Using FLIM/FRET approaches, we show that ERM binding is absolutely required for outside-in L-selectin clustering. The cytoplasmic tail of human L-selectin contains two serine (S) residues at positions 364 and 367, and here we show that they play divergent roles in regulating ERM binding. Phospho-S364 blocks direct interaction with ERM, whereas molecular modeling suggests phospho-S367 likely drives desorption of the L-selectin tail from the inner leaflet of the plasma membrane to potentiate ERM binding. Serine-to-alanine mutagenesis of S367, but not S364, significantly reduced monocyte protrusive behavior in TEM under flow conditions. Our data propose a model whereby L-selectin tail desorption from the inner leaflet of the plasma membrane and ERM binding are two separable steps that collectively regulate protrusive behavior in TEM.

## Introduction

The migration of circulating leukocytes toward extravascular sites of damage or infection is absolutely fundamental to the inflammatory response, and transendothelial migration (TEM) describes the first physical barrier that is breached in this process (1). Chemokine receptors and integrins are major drivers of leukocyte TEM, but little is known about how other receptors participate in this process. L-selectin is a glycan-binding type I transmembrane cell adhesion molecule that plays a well-understood role in regulating cell capture (tethering) and rolling along apically-expressed ligands of inflamed endothelial monolayers (2). L-selectin is constitutively expressed in most circulating leukocytes, and is rapidly cleaved (shed) from the plasma membrane following challenge with formyl peptides, TNF-α, lipopolysaccharide, the complement-derived fragment C5a, or phorbol myristate acetate (PMA)—a potent PKC agonist (3–5). L-selectin shedding occurs at a defined extracellular location, nine amino acids above the plasma membrane (6, 7). Most shedding assays are conducted *in vitro*, using isolated leukocyte subsets (typically monocytes, neutrophils, and naive T-cells). L-selectin shedding in primary human CD14^+^ monocytes has been recently shown to be triggered exclusively during TEM, and not before (8). Moreover, the shedding event is restricted to transmigrating pseudopods in cells captured in mid-TEM (see later).

Rolling leukocytes sense chemokines deposited on the apical aspect of the endothelium, triggering integrin activation and arrest from flow. Upon firm adhesion, leukocytes spread and polarize to establish front-back polarity on the apical aspect of the endothelium. Luminal crawling describes the coordinated protrusion and retraction behavior of leukocytes, sampling and identifying a suitable site to execute TEM. During TEM, leukocytes will protrude a leading edge, most commonly between inter-endothelial junctions, and organize their movement across inflamed endothelial monolayers to successfully enter the subendothelial space. A large amount of the intracellular molecular mechanisms governing TEM has been defined more in endothelial cells than in leukocytes (9). To date, chemokine receptors (10, 11), integrins (12–14), PECAM-1 (15, 16), Junctional Adhesion Molecule-A (17), intercellular adhesion molecule-2 (18), and CD99 (19) have all been shown to regulate leukocyte TEM. Given that the majority of these cell adhesion molecules are concentrated at junctions, there is very little understanding of the spatio-temporal organization of the leukocyte counter-receptors during TEM.

Neutrophils that either lack L-selectin, or express a non-cleavable form of L-selectin, emigrate poorly from cytokine-stimulated cremasteric post-capillary venules (20, 21). Moreover, emigrated neutrophils lacking L-selectin fail to chemotax toward extravascular chemokine gradients *in vivo* (22). Whilst interesting, these *in vivo* observations lack any intracellular mechanistic detail to support the phenotype. More recently, L-selectin has been shown to regulate pseudopod protrusion during human monocyte TEM (8, 23). During TEM, the pool of L-selectin within transmigrating pseudopods makes contact with subendothelial glycans (such as biglycan)—driving it's clustering and ectodomain shedding (8, 24). L-selectin is considered to contribute to outside-in signaling during TEM, specifically within a narrow temporal window: before ectodomain shedding is triggered to shut-down signal transduction. It is noteworthy to mention that clustering of L-selectin in different leukocyte subsets contributes to: β1 and β2 integrin activation (25–27), increased responsiveness to chemokines (28) and increased chemokine receptor expression (29).

Pharmacologic or genetic blockade of L-selectin shedding in primary human monocytes promotes multi-pseudopodial extensions in fully transmigrated cells, culminating in disturbed front-back polarity with reduced persistence in directional migration (8). The underlying molecular mechanism of signal transduction downstream of L-selectin, during TEM, remains poorly understood. Based on previous findings, it is clear that the cytoplasmic tail of L-selectin plays a pivotal role in regulating clustering, ectodomain shedding and signal transduction (2, 30–33). However, L-selectin clustering during TEM has not been interrogated at a mechanistic level. L-selectin binds to a number of intracellular proteins, which include (but are not limited to) calmodulin (CaM) and the ezrin-radixin-moesin (ERM) proteins (31, 34, 35). Earlier studies have shown that the cytoplasmic tail of L-selectin, whilst only 17 amino acids, can form a heterotrimeric complex with CaM and ERM (36). In monocyte cell lines, ligand binding of L-selectin promotes a unique supramolecular assembly of heterotrimeric complexes from adjoining cytoplasmic tails (32, 36). These inter-tail interactions are thought to drive the assembly of an “adhesome-like complex” that is considered unique to L-selectin. The recent reporting of L-selectin binding sequentially to ezrin and then moesin during monocyte TEM (23) suggests L-selectin binding partners are dynamically modulated by reversible mechanisms.

The cytoplasmic tail of human L-selectin possesses two serine residues at positions 364 and 367. Agonists of leukocyte activation (e.g., T-cell receptor and chemoattractant receptor stimulation) promote phosphorylation of Ser^364^ and Ser^367^, via protein kinase C (PKC) isozymes α, τ and θ (3, 37, 38). In transmigrating monocyte pseudopods, phosphorylation of Ser^364^ leads to calmodulin dissociation and subsequent ectodomain shedding of L-selectin (8, 31). Whether ERM also dissociate in response to L-selectin tail phosphorylation has not been addressed. Ezrin and moesin are abundantly expressed in leukocytes, with little to no radixin expression (39). In “resting” (unchallenged) monocytes, L-selectin/ezrin interaction dominates over L-selectin/moesin interaction. Moreover, L-selectin/ezrin interaction is required for protrusive behavior during TEM (23). As TEM proceeds, L-selectin/moesin interaction increases exclusively within transmigrating pseudopods. This exchange is thought to contribute to the clustering of L-selectin prior to ectodomain shedding. Blocking ectodomain shedding of L-selectin leads to its sustained interaction with ezrin, suggesting that moesin acts as a “pro-shedding factor” during TEM. *In vivo* evidence reveals that knocking out moesin in mice leads to net increases in L-selectin surface expression levels, which is not observed in ezrin knockout mice (40, 41). What influences the exchange from ezrin to moesin as TEM proceeds is not understood, but it is tempting to speculate that serine phosphorylation of the L-selectin tail may contribute to this. Ezrin is unique from moesin in that it can bind to the p85 subunit of PI3K (42). It has been hypothesized that ezrin contributes to signaling required to drive protrusive behavior during TEM. In contrast, moesin drives the clustering of L-selectin to prepare it for ectodomain shedding, limiting any further outside-in signaling (23).

Biophysical analyses (43) and *in silico* simulation models (44) have recently hypothesized that the binding of ERM to the L-selectin tail may not be as simple as once thought. When free from its binding partners, the L-selectin tail can interact with the inner leaflet of the plasma membrane through strong electrostatic forces with phospholipids: phosphatidyl serine (PS) (43) and phosphatidylinositol 4,5 bisphosphate (PIP2) (44). Recent studies propose that ERM act to desorb the L-selectin tail from the inner leaflet of the plasma membrane, influenced by local PIP2 concentrations (43, 44). Given that ERM also possess a PIP2-binding site (45), it is likely that they will compete for PIP2 binding to facilitate L-selectin tail desorption from the plasma membrane. Furthermore, it is conceivable that serine phosphorylation of either Ser^364^ or Ser^367^, or both, could facilitate desorption of the L-selectin tail from the plasma membrane by providing a repulsive negative charge cloud. To date, the influence of serine phosphorylation on monocyte protrusive behavior during TEM has not been investigated.

Clustering of L-selectin is known to activate numerous effector responses in different immune cell subtypes. To better understand how L-selectin clustering (and therefore signaling) is regulated during TEM, we engineered the monocyte-like THP-1 cell line to co-express WT or mutant forms of L-selectin that were C-terminally tagged to green or red fluorescent proteins (GFP/RFP). Fluorescence lifetime imaging microscopy (FLIM) was used to quantify Förster resonance energy transfer (FRET) between the GFP and RFP tags, as a direct readout for L-selectin clustering during TEM. As published previously, WT L-selectin reproducibly clustered within transmigrating pseudopods of THP-1 cells captured in mid-TEM. Surprisingly, pharmacologic or genetic blockade of L-selectin shedding completely reversed the distribution of clustered L-selectin to non-transmigrated uropods. Serine-to-alanine mutagenesis of Ser^364^ and Ser^367^ in non-cleavable mutants of L-selectin partially reverted the clustering back to transmigrating pseudopods—implying an important role for cytoplasmic tail serines in regulating the subcellular distribution of L-selectin clustering during TEM. We found that L-selectin/ERM binding is absolutely required for outside-in clustering, and biochemical interactions further showed that phospho-Ser^364^, but not phospho-Ser^367^, directly blocked ERM binding. *In silico* simulation models showed that phospho-Ser^367^, but not phospho-Ser^364^, was sufficient to drive cytoplasmic tail desorption from the inner leaflet of the plasma membrane. These data reveal diametrically opposing roles for serine phosphorylation in regulating ERM binding. Lastly, alanine mutagenesis of Ser^367^ significantly impaired monocyte protrusive behavior during TEM (compared to S364A or WT L-selectin) suggesting an important role for this residue in ERM binding and pseudopod protrusive behavior.

## Results

### ERM Binding Is Absolutely Required for Outside-in Clustering of L-selectin

**Figure 1:**
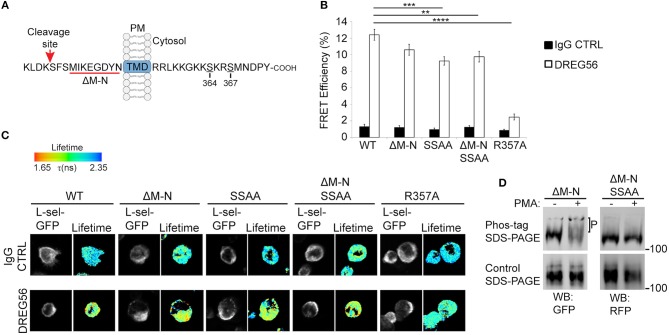
L-selectin/ERM interaction is absolutely required for antibody-mediated clustering (AMC), and Ser^364^ and Ser^367^ are phosphorylated in response to PKC activation. **(A)** Schematic representation of the cleavage site, transmembrane domain (TMD), and cytoplasmic tail of human L-selectin. Red underlined region of the cleavage site denotes the amino acids that are deleted in ΔM-N L-selectin. Amino acid residues Ser^364^ and Ser^367^ are indicated in the cytoplasmic tail of L-selectin. Red arrow points to the position of the cut site of the L-selectin cleavage domain. **(B)** Cells expressing matched levels of L-selectin-GFP/RFP in the following forms: WT, ΔM-N, SSAA, ΔM-N^SSAA^, and R357A were subjected to AMC using Alexa Fluor 647-conjugated DREG56 or isotype-matched control, followed by goat anti-mouse secondary antibody (see Materials and Methods for more detail). Cells were plated onto poly-L-lysine-coated coverslips and prepared for FLIM/FRET analysis (see materials and methods). FLIM was used to measure the FRET efficiency between the GFP/RFP donor/acceptor pairs as a direct function of cytoplasmic tail clustering using. At least 45 individual cells were analyzed over 3 independent clustering assays. **(C)** Representative images are shown for each cell line. The lifetime of fluorescence (Lftm) is expressed in a pseudocolour scale from red (low lifetime with a very high probability of interaction) to blue (high lifetime with a very low probability of interaction). **(D)** THP-1 cell lines expressing ΔM-N L-selectin-GFP/RFP or ΔM-N^SSAA^ L-selectin-GFP/RFP were stimulated with 100 nM PMA for 15 min. Cells were lysed directly in protein loading buffer (containing the reducing agent, dithiothreitol) and resolved onto standard (lower panel) or phos-tag (upper panel) polyacrylamide gels. Antibodies to GFP and RFP were used to, respectively, probe ΔM-N and ΔM-N^SSAA^ on Western blots. “P” represents the electrophoretic mobility shift of phosphorylated ΔM-N, which is not seen in ΔM-N^SSAA^ L-selectin, or in conventional reducing gels. Statistics: One-Way ANOVA Tukey's multiple comparison test. **p ≤ 0.01; ***p ≤ 0.001; ****p ≤ 0.0001.

Historically, antibody-mediated clustering (AMC) of L-selectin has been shown to drive a multitude of responses in different leukocyte subsets. Examples include: the formation of a supramolecular complex between adjoining L-selectin tails, β1 and β2 integrin activation, chemokine receptor expression from intracellular stores, chemokine responsiveness of T-cells and reactive oxygen species production (2, 28, 36, 46–48). These outcomes demonstrate the unequivocal importance of outside-in L-selectin clustering, and its contribution to intracellular signaling. The cytoplasmic tail of L-selectin is known to bind ERM, but the contribution of L-selectin/ERM interaction has never been assessed in respect of AMC. To better understand if Ser^364^ and Ser^367^ within the L-selectin tail contribute to AMC, we mutated them both to alanines (SSAA) in the open reading frames of WT human L-selectin, or a “sheddase-resistant” mutant of L-selectin (hereon called ΔM-N—see Figure 1A and materials and methods for more detail on the mutant). Additionally, arginine at position 357 was mutated to alanine (R357A), which has been shown to block L-selectin/ERM interaction biochemically and in cells (23, 34, 49). All the constructs used in this experiment were cloned into lentiviral vectors containing C-terminally tagged green or red fluorescent protein (GFP/RFP). THP-1 cells (which do not express endogenous L-selectin) were sorted to express matched levels of the L-selectin variants and subjected to AMC as outlined in materials and methods. FLIM was used to quantify FRET between GFP- and RFP-tagged L-selectin in each THP-1 cell line. DREG56 was used to target the lectin domain of L-selectin and secondary antibody was used to further cluster DREG56 to mimic ligand binding and clustering, respectively. FLIM revealed that AMC significantly increased the FRET efficiency in cells expressing WT L-selectin-GFP/RFP from 1.35 to 12.39% (Figures 1B,C). From previous studies (23), we have shown that WT L-selectin/ezrin interaction is dominant in resting cells and implies that ezrin is holding L-selectin in an unclustered configuration (at least to itself). Compared to WT L-selectin, clustering the ERM-binding mutant, R357A L-selectin, lacked any significant increase in FRET efficiency (12.39% [WT] vs. 2.47% [R357A]. Deleting eight amino acids (MIKEGDYN) of L-selectin from the plasma membrane toward the cleavage site renders human L-selectin non-cleavable (ΔM-N) (50). To test the impact of blocking ectodomain shedding on AMC of L-selectin, THP-1 cells expressing ΔM-N L-selectin-GFP/RFP revealed no significant increase in FRET efficiency when cells were at rest, again suggesting that blocking ectodomain shedding of L-selectin did not lead to clustering. However, mutating Ser^364^ and Ser^367^ to alanines in WT and ΔM-N backbone constructs (hereon termed: SSAA and ΔM-N^SSAA^) led to a modest but significant drop in FRET efficiency compared to WT L-selectin (9.25% [SSAA] and 9.76% [ΔM-N^SSAA^] vs. 12.39%[WT]). However, no significant difference in the FRET efficiency of ΔM-N and ΔM-N^SSAA^. An underlying reason for this observation could be that the serine residues in ΔM-N are predominantly dephosphorylated. Indirect assessment of L-selectin serine phosphorylation by phos-tag Western blots revealed that serine phosphorylation was detected only when cells were robustly stimulated with the phorbol ester, PMA (Figure 1D), which is known to drive PKC-dependent phosphorylation of Ser^364^ and Ser^367^ (3, 37). These results corroborate with previous findings that serine phosphorylation of L-selectin is triggered in response to cell-activating stimuli, and, moreover, in the ΔM-N non-cleavable mutant (3). Whilst these data reveal a modest contribution of serine residues in regulating AMC of L-selectin, they highlight an absolute requirement of L-selectin/ERM interaction for outside-in clustering. Moreover, L-selectin [likely through interaction with ezrin, as previously reported Rey-Gallardo et al. (23)] is held in an unclustered configuration in resting cells.

### Ser^364^ and Ser^367^ Orchestrate L-selectin Clustering During Monocyte TEM

**Figure 2:**
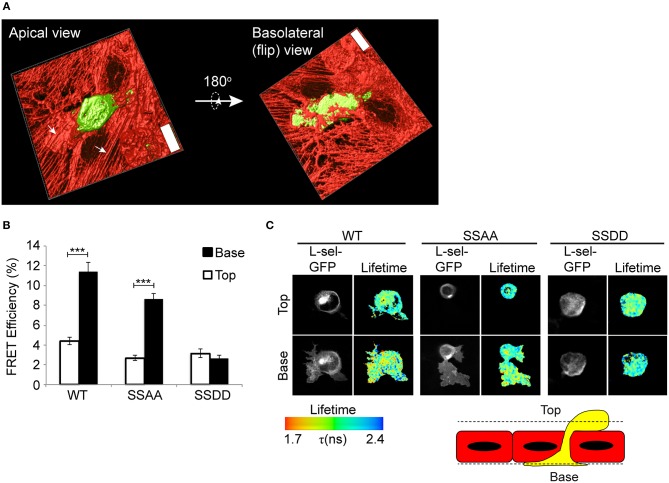
Pseudophosphorylation of L-selectin at Ser^364^ and Ser^367^ blocks clustering of L-selectin within transmigrating pseudopods. **(A)** Line scanning confocal microscopic image of THP-1 cells, stably expressing GFP, captured in mid-TEM—following 15 min of perfusion over TNF-α-activated HUVEC monolayers. Two optical planes are taken to demonstrate that pseudopods are pushing underneath the sub-endothelial space, and that the remaining non-transmigrated part of the cell is round and clearly on the apical aspect of the endothelium. “Top” and “Base,” respectively, represent the apical and basolateral aspect of the endothelium (x63 objective lens, Leica SP5). Scale bar = 24 μm. **(B,C)** THP-1 cells expressing WT, SSAA, or SSDD L-selectin-GFP/RFP were perfused over TNF-α-activated HUVEC and fixed after 15 min of perfusion. This time point is considered when protrusive behavior is maximal and when L-selectin shedding is minimal (8). Lifetime images taken of at least 45 cells and analyzed over 3 independent experiments was quantified and expressed as FRET efficiency. Images representative of three independent experiments, where at least 45 cells of each group was analyzed at two optical sections, Top and Base, as indicated in the cartoon of a yellow monocyte captured in mid-TEM crossing a red endothelium. Individual GFP channel and lifetime images are provided for each cell line. The lifetime of fluorescence is expressed in a pseudocolour scale from red (low lifetime with a very high probability of interaction) to blue (high lifetime with a very low probability of interaction). Statistics: unpaired student *t*-test ***p ≤ 0.001.

Given that AMC of L-selectin does not truly reflect how L-selectin is clustered during TEM, we subjected THP-1 cells to flow assays and asked if Ser^364^ and Ser^367^ contribute to L-selectin clustering in TEM. Our recent work showed that WT L-selectin clusters exclusively within transmigrated pseudopods of THP-1 cells before it is cleaved (8). Moreover, uncleaved full-length L-selectin is present in the transmigrating pseudopods of primary human inflammatory (classical) CD14^+^ human monocytes (8, 23). In this assay, THP-1 cells expressing GFP- and RFP-tagged forms of WT, SSAA or SSDD L-selectin were perfused for 15 min over TNF-α-activated HUVEC and subsequently fixed in mid-TEM (note: at 15 min, protrusive behavior is maximal, but L-selectin shedding is minimal). All mid-transmigrating cells were quantified by FLIM at two distinct optical sections: above and below the endothelial monolayer (termed “Top” and “Base,” respectively, in Figure 2), representing the respective locations of non-transmigrated uropods and transmigrated pseudopods. In agreement with previous data, WT L-selectin clustered exclusively within transmigrated pseudopods (Figure 2). Cell lines expressing SSAA L-selectin-GFP/RFP phenocopied the subcellular distribution of WT L-selectin, suggesting that the pool of WT L-selectin within transmigrated pseudopods likely represents non-phosphorylated L-selectin. Moreover, THP-1 cells expressing phospho-mimicking aspartates (SSDD) L-selectin-GFP/RFP lacked any signs of clustering during TEM—either above or below the endothelium (Figure 2). We can assume that Ser^3^64 and Ser^3^67 are dispensable for L-selectin clustering during TEM, but their phosphorylation completely blocks clustering during TEM.

### Blocking L-selectin Shedding Increases Ser^364^ and Ser^367^ Phosphorylation and Subcellular Organization of Clustering, Specifically During TEM

**Figure 3:**
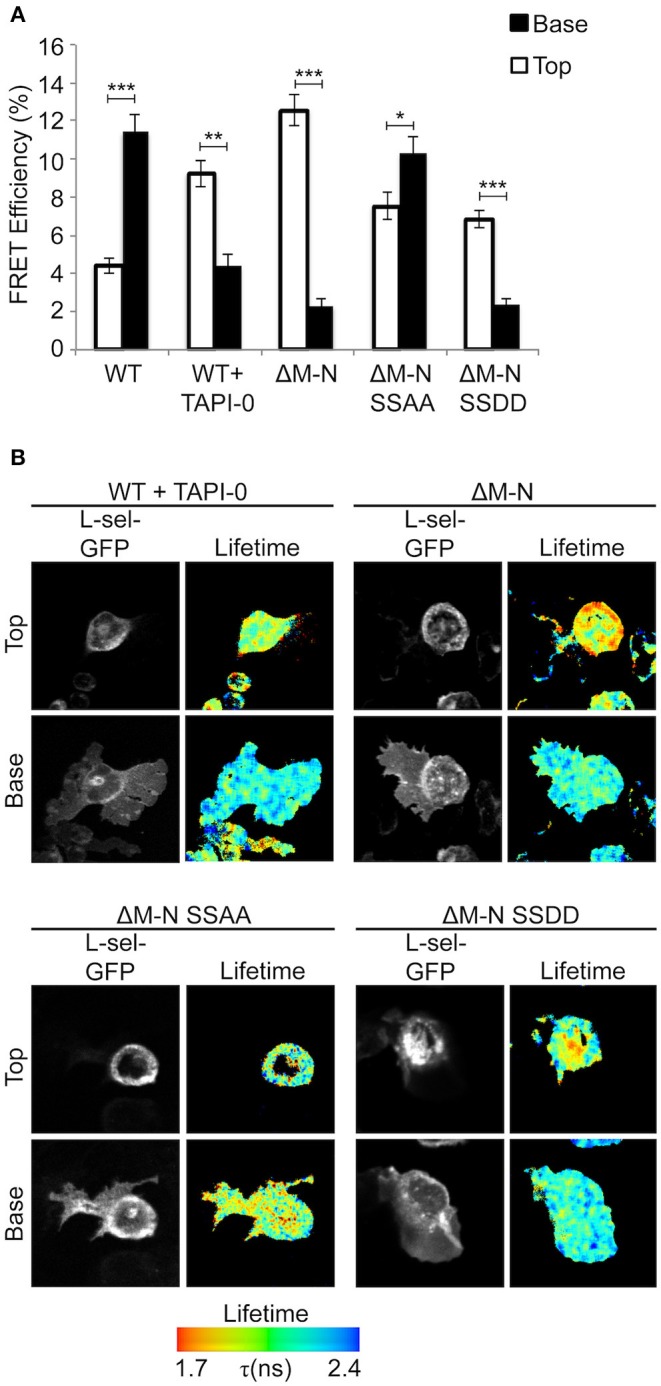
Blocking ectodomain shedding of L-selectin during TEM alters the subcellular distribution of clustering through increased phosphorylation of Ser^364^ and Ser^367^. Cells expressing WT L-selectin-GFP/RFP were treated for 30 min with 10 μM TAPI-0 and subsequently perfused over TNF-activated HUVEC monolayers for 15 min prior to fixation and analysis for FRET by FLIM (see Materials and Methods for more detail). Note that 10 μM TAPI-0 was supplemented in the perfusion medium during the flow experiment. Other cell lines expressing non-cleavable mutants were also perfused under similar conditions, but without 10 μM TAPI-0. **(A)** FLIM was used to calculate the % FRET efficiency for each cell line expressing L-selectin-GFP/RFP, both in non-transmigrated uropods (‘Top,” white bars) and transmigrated pseudopods (“Base,” black bars). **(B)** Images representative of three independent experiments, where at least 45 cells of each group were analyzed at two optical sections–non-transmigrated uropods (Top) and transmigrated pseudopods (Base). GFP fluorescence channel and lifetime images are provided for each cell line. The lifetime of fluorescence is expressed in a pseudocolour scale from red (low lifetime with a very high probability of interaction) to blue (high lifetime with a very low probability of interaction). Statistics: unpaired student *t*-test: *p ≤ 0.05; **p ≤ 0.01; ***p ≤ 0.001.

We have previously shown that blocking ectodomain shedding of L-selectin drives THP-1 cells and monocytes to produce multiple pseudopodial extensions in TEM (8). We therefore asked if L-selectin clustering was causal to the multi-pseudopodial extension phenotype. THP-1 cells expressing WT L-selectin-GFP/RFP were first challenged with 10 μM of the metalloproteinase inhibitor, TNF-alpha proteinase inhibitor-0 (TAPI-0), to block ectodomain shedding during the 15 min period for TEM. Quantification of transmigrating cells by FRET/FLIM revealed that a large majority of L-selectin clustering had relocated from transmigrated pseudopods to non-transmigrated uropods (Figure 3). This profound switch in subcellular organization was phenocopied in THP-1 cells expressing ΔM-N L-selectin-GFP/RFP, strongly suggesting that 10 μM TAPI-0 was directly impacting the L-selectin sheddase, a disintegrin and metalloproteinase 17 (ADAM17), without any obvious off-target effect. To determine if serines Ser^364^ and Ser^367^ played a role in the response, THP-1 cells expressing ΔM-N^SSAA^-GFP/RFP were perfused and analyzed under similar experimental conditions. In contrast to the ΔM-N cell line, the ΔM-N^SSAA^ mutant cell line partially reverted the subcellular organization of L-selectin clustering toward that of WT L-selectin (Figure 3). These results suggest that the serine residues play a major role in orchestrating the subcellular distribution of non-cleavable L-selectin clustering during TEM. Engineering the SSDD phospho-mimicking mutation into the non-cleavable ΔM-N L-selectin-GFP/RFP backbone (hereon called ΔM-N^SSDD^) allowed us to interrogate the clustering of this mutant during TEM. FRET/FLIM analysis revealed that the ΔM-N^SSDD^-GFP/RFP mutant faithfully phenocopied the clustering distribution of ΔM-N L-selectin, strongly suggesting that serine phosphorylation is driving L-selectin clustering in to non-transmigrated uropods. Taken together, the pool of ΔM-N L-selectin within the non-transmigrated uropod is likely to exist in a predominantly serine phosphorylated form. Although the extent of AMC in cells expressing ΔM-N or ΔM-N^SSAA^ was completely indistinguishable (Figure 1B), their subcellular distribution of clustering during TEM was profoundly different (Figure 3). These data highlight the impact that blocking ectodomain shedding of L-selectin has on its subcellular organization in clustering during TEM. Moreover, it highlights the essential role that serine residues play in orchestrating the subcellular distribution of L-selectin clustering during TEM.

### Phosphorylation of Ser^364^ Directly Interferes With FERM Binding

**Figure 4:**
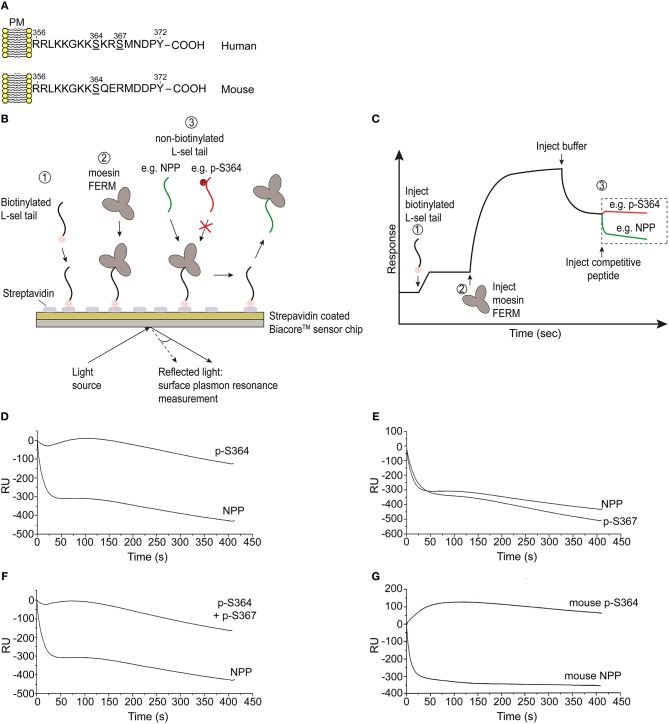
Phosphorylation of Ser^364^, but not Ser^367^, abrogates FERM domain binding *in vitro*. **(A)** Amino acid sequences corresponding to the cytoplasmic tails of human and mouse L-selectin. Serine residues are underlined in each linear sequence. Note that mouse L-selectin carries a single serine residue at position 364. **(B)** Outline of the competition assay, depicted in 3 steps: 1 = immobilization of N-terminally biotinylated peptide, corresponding to the tail of L-selectin, on to the streptavidin-coated sensorchip. 2 = injection of 5 μM soluble moesin FERM domain, which binds to the immobilized L-selectin tail peptide. 3 = injection of 100 μM soluble non-biotinylated L-selectin tail peptide (depicted in green) leads to efficient competition. In contrast, serine phosphorylation of the L-selectin tail that blocks FERM interaction will act as a poor competitor (as depicted by the red colored tail peptide). **(C)** Schematic of a typical trace, indicating the various steps in **(B)**, which we have reported elsewhere (34). Importantly, the traces represented in red and green are the profiles that represent the timelines of competitor peptide injections. **(D)** Competition profiles of phospho-Ser^364^ (p-S364) and non-phosphorylated peptide (NPP). **(E)** Competition profiles of phospho-Ser^367^ (p-S367) and NPP. **(F)** Competition profiles of double-phosphorylated peptide: p-S364 and p-S367 alongside NPP. **(G)** Competition profiles of mouse p-S364 and mouse NPP. Each graph represents one of three independent experiments.

Given that L-selectin/ERM interaction is absolutely essential for L-selectin clustering, and that serine phosphorylation is regulating L-selectin clustering during TEM, we next questioned if serine phosphorylation directly regulates ERM binding. Multiple biochemical approaches have confirmed that the N-terminal domain of ERM (hereon called: four point one ezrin radixin moesin—FERM) interacts with peptides corresponding to the tail of L-selectin (34, 36, 43, 49). The high level of amino acid identity between moesin and ezrin FERM (≥85%) means that biochemical approaches cannot discriminate differences in binding of L-selectin with the FERM domains of either ezrin or moesin. However, such experiments can reliably inform whether serine phosphorylation of the L-selectin tail impacts FERM binding. A series of non-phosphorylated (NPP) and phospho-peptides corresponding to the 17 amino acid tail of L-selectin were synthesized and used in competition assays, which we have previously reported (34) (see Figures 4B,C, and materials and methods for details). In short, biotinylated NPP corresponding to the human L-selectin tail was immobilized onto a streptavidin-coated biosensor chip (for surface plasmon resonance studies). Subsequent injection of moesin FERM into the biosensor chip enabled a stable complex to form with chip-immobilized L-selectin tail. The competitive capacity of peptides to disrupt the chip-immobilized L-selectin/FERM complex would shed light on their importance in regulating ERM binding in cells. Phospho-peptides with strong competitive capacity were deemed to carry non-essential phospho-serines that would not block ERM binding in cells. In contrast, phospho-peptides that were weakly competitive were deemed to carry phospho-serines that would block ERM binding in cells. As expected, saturating the biosensor chip with 100 μM of NPP led to a sharp drop in response units (RU) at the biosensor chip, indicating strong competition of chip-immobilized moesin FERM (Figure 4D). However, injection of 100 μM peptide, specifically phosphorylated at Ser^364^ (p-S364), failed to compete-off the chip-immobilized moesin/FERM complex (Figure 4D). In contrast, phospho-Ser^367^ (p-S367) peptide strongly competed chip-immobilized moesin FERM to the same degree as the NPP and suggested phospho-Ser^367^ would not interfere with FERM binding in cells (Figure 4E). The competition profile of phospho-peptide containing both phospho-Ser^364^ and phospho-Ser^367^ was similar to the phospho-peptide profile of phospho-Ser^364^ (Figure 4F), suggesting that phospho-Ser^367^ did not hinder the ability of phospho-Ser^364^ to block FERM interaction. The tail of mouse L-selectin contains a single serine residue at position 364, suggesting possible conserved mechanisms with human L-selectin at this site (Figure 4A). Indeed, phospho-Ser^364^ peptide of mouse L-selectin also failed to compete the biosensor chip-immobilized FERM/L-selectin complex (Figure 4G). Taken together, these data suggest that phosphorylation of L-selectin at Ser^364^, but not Ser^367^, abrogates FERM binding in both mice and humans.

### Molecular Dynamics Implies phospho-Ser^367^ Desorbs the L-selectin Tail From the Inner Leaflet of the Plasma Membrane

As phospho-Ser^367^ didn't block the binding of either calmodulin (8) or ERM proteins (Figure 4), we questioned whether it could regulate desorption of the L-selectin tail from the inner leaflet of the plasma membrane. Biophysical approaches and molecular dynamics (MD) suggest that the tail of L-selectin forms strong electrostatic interaction with phospholipids, such as phosphatidyl serine (PS) and phosphatidylinositol-4,5, bis-phosphate (PIP2), which are both enriched in the inner leaflet of the plasma membrane (43, 44). Binding of L-selectin to lipid bilayers containing PIP2 or PS precludes calmodulin binding, raising the question if serine phosphorylation of the L-selectin tail can drive cytoplasmic tail desorption.

**Figure 5:**
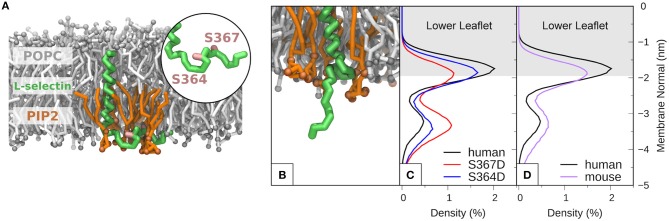
Molecular modeling reveals that phosphorylation of S367 in human L-selectin leads to desorption of the tail from the inner leaflet of the plasma membrane. **(A)** Snapshot of non-phosphorylated human L-selectin embedded in a POPC bilayer with 6% PIP2 in the lower leaflet. L-selectin backbone beads are shown in green with Ser^364^ and Ser^367^ marked in pink (see circular inset and Supplementary Video 1). POPC and PIP2 lipids are depicted in gray and orange, respectively. Solvent and ion molecules are omitted for clarity. **(B)** Snapshot of L-selectin S367D, displaying increased electrostatic repulsion between L-selectin tail residues and PIP2 lipids thus promoting desorption from the lower leaflet. The color code in **(B)** is the same as in **(A)**. **(C)** Density distributions of Y372 of L-selectin with respect to the lipid bilayer in non-phosphorylated (black line), S364D (blue line) and S367D (red line) L-selectin. **(D)** Direct comparison of density distribution profiles between non-phosphorylated human (black line) and mouse (purple line) L-selectin tail.

MD of human L-selectin in 1-palmitoyl-2-oleoyl-sn-glycero-3-phosphatidylcholine (POPC) bilayer containing 6% PIP2 lipids randomly distributed in the lower leaflet showed agreement with previous MD simulations (44). Specifically, PIP2 lipids surrounded the L-selectin transmembrane domain, where Ser^364^ and Ser^367^ were observed to intercalate amid the PIP2 headgroups (see Supplementary Video 1 and Figure 5A). Engineering the S367D mutation into the L-selectin tail promoted desorption and extension of the L-selectin tail (see Figure 5B). By tracing the position of the C-terminal tyrosine residue at position 372 (Y372) in S367D, S364D, and non-phosphorylated L-selectin, we could quantify its density distribution over a 12 μs simulation period (see materials and methods for more details). Compared to non-phosphorylated L-selectin, the density distribution of Tyr^372^ in S367D shifted away from the inner leaflet toward the cytosol (compare red and black profiles in Figure 5C). A reduced effect was observed for S364D L-selectin, with density distribution profiles between non-phosphorylated and S367D L-selectin tail (compare blue and black profiles in Figure 5C). As Ser^367^ is missing from mouse L-selectin tail, MD was performed on this species to determine if desorption could occur without Ser^367^ phosphorylation. In mouse L-selectin, Asn^369^ is replaced by Asp, thus decreasing the net charge of mouse L-selectin by 1e compared to non-phosphorylated human L-selectin. As shown in Figure 5D, side-by-side comparison of density profiles corresponding to non-phosphorylated mouse and human L-selectin showed a significant shift of Tyr^372^ away from the bilayer in mouse L-selectin, closely resembling the S364D L-selectin profile in Figure 5C. These data suggest that the mouse L-selectin tail is less adsorbed to the inner leaflet of the plasma membrane than human L-selectin, which may support ERM binding more readily during TEM. Whilst these data provide insight into molecular mechanism regarding tail desorption by phospho-Ser367, they remain speculative until proven by other experimental means.

### S367A L-selectin Significantly Reduces the Protrusive Behavior of THP-1 Cells Undergoing TEM

**Figure 6:**
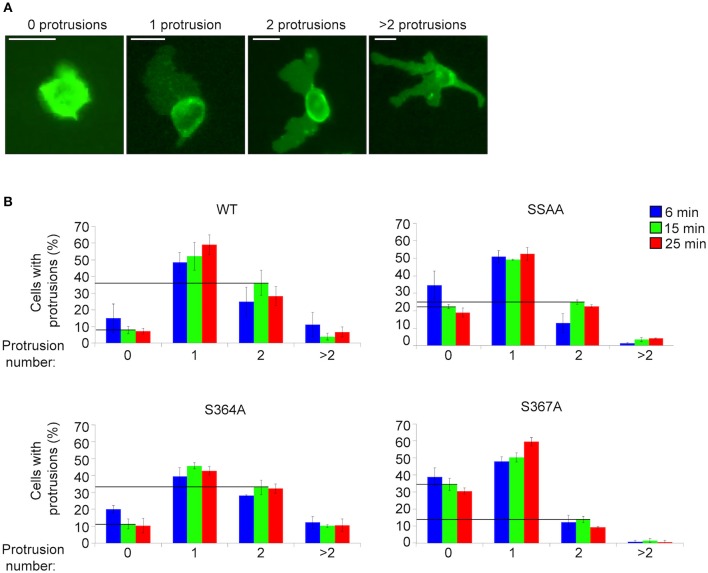
S367A L-selectin significantly reduces pseudopod protrusive behavior in TEM. Each cell line expressing either WT or mutant L-selectin was perfused over TNF-α-activated HUVEC for a period of 25 min. **(A)** The number of protrusions formed over this period was scored as: zero, one, two, or >2. **(B)** The percentages of cells bearing these protrusions were scored at specific time points: 6 min (blue bars), 15 min (green bars), or 25 min (red bars). Supplementary Videos 2, 3, 4 provide examples of cells producing a range of protrusions as TEM proceeds. Data represent SEM of 3 fields of view per flow experiment, conducted on three separate occasions, and at least 180 cells analyzed per group. Values of the vertical lines indicate the differences in protrusion number (specifically “zero” and “two”) corresponding to each of the cell lines, for which statistical significance is shown in Table 1.

MD modeling strongly suggested that phosphorylation of Ser^367^ regulates desorption of the L-selectin tail from the inner leaflet of the plasma membrane (Figure 5). Moreover, we have previously published significantly reduced interaction of calmodulin with S367A L-selectin in transmigrating pseudopods. We therefore hypothesized that S367A would hinder desorption of the L-selectin tail, reducing ERM interaction and monocyte protrusive behavior during TEM. THP-1 cells expressing S364A, S367A, or SSAA L-selectin-GFP were therefore subjected to flow assays and their protrusion dynamics assessed over a 25 min period (see Figure 6A and associated Supplementary Videos 2, 3, 4) as previously described (23). Transmigrating cells were scored as having zero, one, two or multiple protrusions over 3 different time points (6, 15, and 25 min). THP-1 cells expressing L-selectin S367A possessed the fewest protrusions over the recorded period, differing significantly from WT and S364A L-selectin-expressing cell lines (see Figure 6B and Table 1). Moreover, 34.4% of S367A cells did not possess protrusions at the 15 min time point, compared with only 7.9% of WT cells with zero protrusions (Table 1 and Figure 6B). In contrast, whilst 36.2% of WT cells presented two protrusions at the 15 min time point, only 13.7% of S367A cells presented two protrusions at this time point (Table 1 and Figure 6B). We noted that the S364A mutant produced a profile of protrusive activity that was similar to cells expressing WT L-selectin. Our data suggest that the S364A mutation does not impact Ser^367^ phosphorylation, cytoplasmic tail desorption and ERM binding. As anticipated, the SSAA mutant cell line phenocopied more the S367A than the S364A cell line. It is possible that the S367A mutant can resist ERM binding more potently than the SSAA mutant—as phosphorylation at Ser^364^ remains intact. Alternatively, in the SSAA mutant, whilst this might reduce the capacity to desorb from the inner leaflet of the plasma membrane, its ability to retain ERM binding would be much higher due to a lack of phosphorylation at position 364. We conclude that Ser^367^ is an important residue for monocyte protrusion in TEM, by regulating cytoplasmic tail desorption and allowing subsequent ERM binding.

**Table 1:** Comparison of protrusive behavior between THP-1 cell lines, expressing WT and mutant L-selectin, exposed to hydrodynamic shear stress.

	**CELL LINE**			
	**WT**	**S364A**	**S367A**	**SSAA**
0 Protrusions	7.9	11.3	34.4^***^	22.4^**^
2 Protrusions	36.2	32.9	13.7^**^	24.9

*Values are taken from the 15 min time point represented in Figure 6 (vertical lines). Data represent mean of 3 fields of view per flow experiment, conducted on three separate occasions, with at least 180 cells analyzed per group. Statistics: One-Way ANOVA Tukey's multiple comparison test*:

** *p ≤ 0.01*,

*** *p ≤ 0.001*.

## Discussion

Until recently, WT and non-cleavable mutants of L-selectin [such as LΔP (51), ΔM-N (50) and L(E) (21)] were not considered to transduce different intracellular signals. In this report, we have exposed profound differences in how ΔM-N L-selectin is clustered during TEM—and how this could contribute to the altered protrusive behavior in TEM. Blocking L-selectin shedding in primary human CD14^+^ (classical “inflammatory”) monocytes disturbs front-back polarity in cells that have entered the subendothelial space, post-TEM (8). CD14^+^ inflammatory monocytes are known to drive atherosclerosis and increase cardiovascular events in humans (52–54). If blocking L-selectin shedding can bring improved outcome within a specific disease setting, then understanding the intracellular signals that are transduced downstream of non-cleavable L-selectin warrants further investigation. In support of this view, one recent study has demonstrated that blocking L-selectin shedding in cytotoxic T-cells confers viral protection in mucosal- and visceral-infected organs (55).

WT and ΔM-N L-selectin bind differently to ezrin and moesin during TEM, suggesting that altered signal transduction could precipitate as a direct consequence of skewed ERM binding behavior. Specifically, ezrin remains bound to ΔM-N L-selectin over a 25 min period of analysis during TEM (23). In contrast, the subcellular distribution of WT L-selectin changes over time during TEM: at 6 min, WT L-selectin localizes with ezrin in transmigrated pseudopods and uropods. In contrast, by 25 min, WT L-selectin remains bound to ezrin at the non-transmigrated uropod, but switches affiliation with moesin within transmigrating pseudopods. Ezrin and moesin differ in their capacity to interact with PI3K, which could explain why ΔM-N cells have higher protrusive activity during TEM—as pseudopod formation is Rac-dependent, and PI3K can lie upstream of Rac activation (56–58). In support of these findings, ΔM-N R357A L-selectin (i.e., a non-cleavable L-selectin that cannot bind ERM proteins) was shown to possess significantly fewer multi-pseudopodial extensions in THP-1 cells undergoing TEM (23).

**Figure 7:**
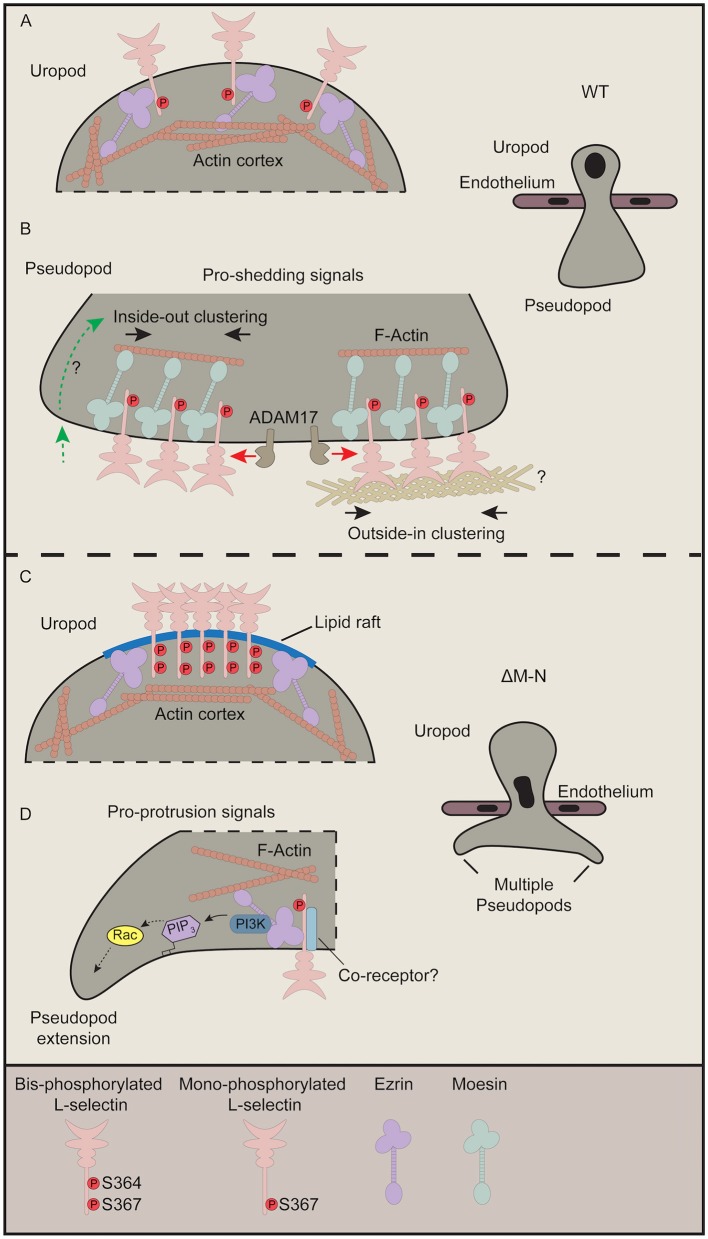
Current perspective on how L-selectin clustering during TEM regulates monocyte protrusive behavior. This figure pieces together data from our recently published work (8, 23) with our data from this study. **(A,B)** In cells expressing WT L-selectin, clustering is only witnessed within pseudopods of transmigrating cells. Although ezrin interacts with WT L-selectin in early TEM to mediate protrusion (not shown), this interaction is rapidly swapped-out by moesin to drive clustering (23). Moesin-driven clustering is a pre-requisite for L-selectin shedding, and so the balance of L-selectin/ezrin and L-selectin/moesin interaction is what ultimately regulates the protrusive activity in TEM. Our FLIM/FRET analysis of SSAA L-selectin suggests that the clustered L-selectin within transmigrated pseudopods is non-phosphorylated (Figure 2). Moreover, given that the protrusive behavior of S364A L-selectin phenocopies more WT than S367A cells (Figure 6 and Table 1), our data also suggest that phospho-Ser^364^ inhibits clustering but phospho-Ser^367^ doesn't interfere with L-selectin clustering. **(B)** There are two proposed modes by which WT L-selectin is clustered within transmigrating pseudopods. The first is via inside-out signals (left hand side—green dotted line with green arrow). The question mark implies that these signals are undefined but could be derived from integrin-mediated clustering and signaling, and/or chemokine receptor signaling. Secondly, classic outside-in clustering is known to drive the clustering in this subcellular region. Indeed, we have shown that isolated THP-1 cells expressing WT L-selectin-GFP/RFP can cluster when seeded onto immobilized biglycan, which was not observed in cells expressing ΔM-N L-selectin-GFP/RFP (8). In these cases, it is possible that Ser^367^ is constitutively phosphorylated but not Ser^364^. To reduce the complexity of our proposed model, we have included a separate model for phospho-cycling at Ser^364^ and Ser^367^ in the L-selectin tail in Figure 8. **(C)** We speculate that non-cleavable ΔM-N L-selectin clusters at the uropod, by default, into lipid rafts due to bis-phosphorylation of Ser364 and Ser367 and uncoupling from ERM. The coalescence of L-selectin within lipid rafts may give rise to false-positive increases in FRET efficiency exclusively within the uropod (Figure 3). We have previously shown a strict preference of interaction for ΔM-N L-selectin with ezrin during early and late TEM (23). Given that ezrin is known to associate with lipid rafts in other immune cells (59), this may also involve the stochastic interactions between ΔM-N L-selectin and ezrin in this microdomain. **(D)** The pool of ΔM-N L-selectin within transmigrated pseudopods does not appear to co-cluster with itself, yet interacts selectively with ezrin to drive multi-pseudopodial extensions. We cannot exclude the possibility that ΔM-N L-selectin co-clusters with another as yet unidentified co-receptor (drawn in light blue), and that this co-clustering is essential for driving the multi-pseudopod phenotype. We have previously shown that ezrin interacts with L-selectin in both uropods and pseudopods (23). We suggest that L-selectin/ezrin complexes are not co-clustered, and, in this configuration, bestow cells with a higher pro-invasive potential during TEM. Given that ezrin can selectively interact with PI3K (42), we believe that this unique coupling could act as a major driver of the multi-pseudopod phenotype.

Based on recent data (23) and data from this report, we propose that the ezrin-bound to ΔM-N L-selectin within transmigrating pseudopods is not clustered. We previously demonstrated that “GFP spots,” representing full-length clustered WT L-selectin-GFP, was significantly higher in transmigrating pseudopods of WT THP-1 cells than in transmigrating pseudopods of ΔM-N THP-1 cells (8). However, the FRET/FLIM analysis in this study has definitively confirmed that ΔM-N L-selectin does not cluster in ≤ 10 nm distances in transmigrating pseudopods. One can conclude that ΔM-N L-selectin in transmigrating pseudopods is either monomeric or co-clusters with an as yet unidentified receptor during TEM. A defining feature of L-selectin/ezrin interaction might be to transduce intracellular signals in its monomeric form. In contrast, L-selectin/moesin interaction is thought to drive clustering just prior to ectodomain shedding. Figure 7 provides a summary of how we hypothesize ERM interacting with WT and ΔM-N L-selectin during TEM.

During TEM, ΔM-N L-selectin is known to constitutively associate with ezrin in THP-1 cells. Moreover, this interaction resides both within transmigrating pseudopods and non-transmigrated uropods (23). Given that the subcellular distribution of ΔM-N^SSDD^ phenocopies that of ΔM-N L-selectin strongly implies that ΔM-N within non-transmigrated uropods is phosphorylated at positions Ser^364^ and Ser^367^. In support of this view, the ΔM-N^SSAA^ mutant reverses the distribution of clustering from non-transmigrated uropods back to transmigrated pseudopods. Moreover, these data show that Ser^364^ and Ser^367^ play important roles in orchestrating the subcellular organization of L-selectin clustering of non-cleavable L-selectin during TEM. Given that WT L-selectin is phenocopied by SSAA L-selectin suggests that clustering within transmigrating pseudopods exists in a predominantly non-phosphorylated form. Indeed, no clustering is observed in cells expressing the SSDD mutant of L-selectin. We can now build on our previous findings (23) to propose that the interaction of ezrin with ΔM-N in the non-transmigrated uropod is likely to be a false positive observation (see more details later), as, biochemically, we have shown that bis-phosphorylated L-selectin blocks interaction with moesin FERM (Figure 4).

The results obtained from AMC of WT and mutant L-selectin indicate that caution should be taken in corroborating these outcomes with clustering induced in bi-cellular systems, such as in TEM. That differences in clustering of WT and ΔM-N L-selectin were modest in AMC experiments but completely different in TEM strongly suggests influences beyond classic outside-in clustering must be in operation. AMC exclusively explores the outside-in mode of clustering, where it seems that blocking L-selectin shedding has very little impact in this regard. The inside-out mechanisms, however, which are likely to be triggered during TEM, can be influenced by numerous input signals: chemokine receptors, integrin clustering and signaling, and mechanotransduction imposed by hydrodynamic shear stress. Chemoattractant stimulation is sufficient to drive serine phosphorylation in L-selectin in numerous different leukocyte subsets (3), suggesting that this event alone will impact on the binding behavior between L-selectin and ERM/calmodulin, and therefore clustering/ectodomain shedding during TEM. Future experiments using “3-way FRET” may shed light on the sequential binding between L-selectin and its binding partners during TEM, which is currently beyond the scope of this report.

Blocking L-selectin shedding revealed a high level of clustered L-selectin in non-transmigrated uropods. We believe that this localization of L-selectin is not driven through direct contact with a luminal ligand. It is more likely that serine phosphorylation of the L-selectin tail drives its localization into specialized membrane microdomains, such as lipid rafts. Accumulation of ΔM-N L-selectin into lipid raft microdomains is likely to increase the propensity for ligand-independent clustering. Indeed, a fraction of L-selectin has been shown to localize in lipid rafts of resting immune cells (60). Polarized T-cells are characterized as possessing two different lipid raft domains: GM1 at the uropod and GM3 at the pseudopod (61). GM1 is present on CD14^+^ inflammatory monocytes and THP-1 cells (62, 63). We would suggest that blocking L-selectin shedding during TEM increases Ser^364^ and Ser^367^ phosphorylation, followed by relocalization into GM1 rafts during TEM. Although these observations are made exclusively when L-selectin shedding is blocked, it convincingly demonstrates the impact that blocking ectodomain shedding of L-selectin has on cell surface localization, clustering, intracellular signaling and protrusive behavior during TEM. From these data, we suggest ezrin interacts with ΔM-N L-selectin indirectly in uropods and directly in transmigrating pseudopods (see Figure 7). Ezrin is known to associate with lipid rafts in leukocytes (59), whereas moesin is excluded from lipid rafts (64), so it is possible that bis-phosphorylated L-selectin is interacting stochastically with ezrin within this microdomain.

**Figure 8:**
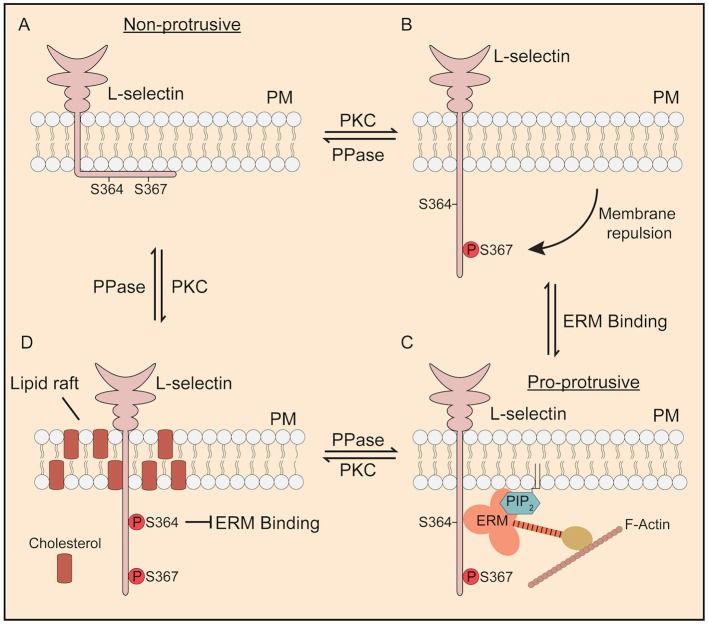
Proposed model of how phosphorylation of Ser^364^ and Ser^367^ modulate L-selectin/ERM interaction during TEM. **(A)** In its non-phosphorylated state, the L-selectin tail forms strong electrostatic interactions with phospholipids such as phosphatidyl serine (43) and PIP2 (44) that are enriched within the inner leaflet of the plasma membrane. Cells expressing 367A, but not S364A, are less permissive for pseudopod protrusion in TEM. **(B)** PKC isozymes are known to phosphorylate the tail of L-selectin (37). Phosphorylation of Ser^367^, but not Ser^364^, drives repulsion of the L-selectin tail from the inner leaflet of the plasma membrane. **(C)** Phospho-Ser367 encourages ERM binding, through increased propensity for plasma membrane desorption. Our model would suggest that, in this configuration, L-selectin bound to ezrin would possess pro-protrusive properties (see Figure 7 for details on how ezrin regulates protrusion). **(D)** Finally, we have previously shown that p-Ser^364^ drives the dissociation of calmodulin to promote ectodomain shedding of L-selectin. However, in non-cleavable ΔM-N L-selectin, bis-phosphorylated L-selectin would block binding to ERM and drive its accumulation into lipid rafts. We believe all of these steps to be reversed by the action of serine/threonine phosphatases (PPase), which has yet to be identified.

Finally, MD has enabled us to explore the possible contribution of Ser^367^ in regulating desorption of the L-selectin tail from the inner leaflet of the plasma membrane. We believe that phospho-cycling of Ser^364^ and Ser^367^ collectively contribute to how L-selectin/ERM binding is regulated to drive pseudopod protrusion in TEM. Figure 8 provides a summary by which these mechanisms are thought to dynamically regulate pseudopod protrusion in TEM. Currently, the MD experiments are purely speculative and will require validation by other experimental techniques. For example, the combination of phospho-specific antibodies (which are currently commercially unavailable) alongside super-resolution microscopy will provide a better understanding of how these two serine residues are regulated in space and time in primary human leukocytes undergoing TEM. Other techniques, such as the biophysical approaches that first conceived the phenomenon of cytoplasmic tail desorption for L-selectin (43) can also be performed to validate the MD data.

## Materials and Methods

### Chemicals and Antibodies

All chemicals and solutions were purchased from Sigma Aldrich, unless otherwise stated. DREG56 was purchased from Santa Cruz Biotechnologies. Anti-GFP and anti-RFP antibodies were purchased from Chromotek. IRDye 680RD and IRDye 800CW (Oddesy) were used as secondary antibodies for LI-COR imaging of Western blots.

### Cell Lines and Culture

The generation of WT and mutant L-selectin lines have been described previously (8), including the cloning strategies to generate mutant lines. All cell lines were cultured at 37°C in medium containing 5% CO_2_ under humidifying conditions. The THP-1 monocytic cell line was purchased from the American Type Culture Collection (LGC Standards) and passaged in RPMI medium containing 10% heat-denatured fetal calf serum (FCS), 1% antibiotics (penicillin/streptomycin) and 50 μM β-mercaptoethanol. Cells were tested negative for mycoplasma. HUVECs were purchased from Lonza and maintained in endothelial cell growth medium (EGM-2) supplemented with growth factors and antibiotics provided within their “bullet kits.” Cells were initially expanded for six or fewer passages, and were harvested and stored in liquid nitrogen for final use in flow assays or western blotting. Confluent HUVECs were disaggregated with trypsin/EDTA solution and seeded onto 10 μg/ml bovine-derived fibronectin. HEK 293T cells were used for lentiviral production and were a kind gift from Yolanda Calle, University of Roehampton, London, UK. Cells were routinely passaged at a 1:3 ratio on the third day. This “splitting” activity maintained an optimal cell density at 0.5 ×10^6^ cells per mL.

### Lentiviral Expression Constructs

The open reading frame for WT and ΔM-N L-selectin was cloned into lentiviral vectors as previously described (8). The pHR'SIN-SEW lentiviral backbone vector was provided by Adrian Thrasher from the Institute of Child Health (University College London, United Kingdom). Constructs were C-terminally tagged with either enhanced green fluorescent protein (eGFP) or monomeric (m)Cherry (a close spectral variant of RFP). For ease of nomenclature, constructs were labeled as “GFP” or “RFP.”

Mutagenesis of the serine to alanine or aspartate residues was conducted using a QuikChange Site-Directed Mutagenesis Kit (Agilent). The following forward (Fwd) and reverse (Rev) primers were used:

***S364A***

Fwd GATTAAAAAAAGGCAAGAAAGCCAAGAGAAGTATGAATGACC.

Rev GGTCATTCATACTTCTCTTGGCTTTCTTGCCTTTTTTTAATC.

***S367A***

Fwd GGCAAGAAATCCAAGAGAGCTATGAATGACCCATATCAC.

Rev GTGATATGGGTCATTCATAGCTCTCTTGGATTTCTTGCC.

***SSAA***

Fwd GGCAAGAAAGCCAAGAGAGCTATGAATGACCCATATCAC.

Rev GTGATATGGGTCATTCATAGCTCTCTTGGCTTTCTTGCC.

***SSDD***

Fwd GGCAAGAAAGACAAGAGAGATATGAATGACCCATATCAC.

Rev GTGATATGGGTCATTCATATCTCTCTTGTCTTTCTTGCC.

***R357A***

Fwd GGCATTTATCATTTGGCTGGCAAGGGCATTAAAAAAAGGCA AGAAATCCAAG.

Rev CTTGGATTTCTTGCCTTTTTTTAATGCCCTTGCCAGCCAAATGATAAATGCC.

### Antibody-Mediated Clustering (AMC)

THP-1 cells were adjusted the night before AMC to a density of 0.5 ×10^6^ cells per mL. On the same day, 13 mm diameter glass coverslips (thickness “1”) were placed into the base of a 24 well dish, spotted with 100 μL of poly-L-lysine (PLL) to immobilize according to manufacturer's instruction. On the day of the assay, THP-1 cells were counted and adjusted to a density of 1 ×10^6^ per mL in 500 μL containing antibody labeling buffer (RPMI culture medium containing FcR block [Miltenyi Biotec]). Cells were labeled with 2 μg per mL DREG56 for 30 min at 4°C, followed by washing (by centrifugation at 300 *g* and resuspension) in ice cold culture media to remove excess unbound antibody and then incubated back in ice cold labeling buffer containing secondary antibody conjugated to Alexa Fluor 663 (Thermo Fisher) for a further 30 min at 4°C. Cells were then washed twice in ice cold neat RPMI and resuspended to 100 μL of neat ice cold RPMI before 80 μL of the cell suspension was seeded onto 13 mm diameter glass coverslips (thickness = “1”), which were pre-coated with PLL-coated the night before. The seeded cells were placed into a humidified cell culture incubator at 37°C and 5% CO_2_ for 10 min to drive clustering. Adhered cells were flooded in excess 4% paraformaldehyde and fixed for 15 min at room temperature. Fixed coverslips were washed 3 times in PBS and then treated in 10 mg/mL of Sodium Borohydride dissolved in phosphate buffered saline for 10 min at room temperature (to eliminate autofluorescence and enhance signal to noise ratios). Coverslips were subsequently washed in PBS to remove sodium borohydride and mounted using DAKO mounting medium.

### SDS-PAGE, Phos-tag™ SDS-PAGE, and Immunoblotting

SDS-PAGE was performed with 5% polyacrylamide gels. Proteins were transferred to 0.45 μm nitrocellulose membranes (Amersham™) using a wet blotting apparatus. Phos-tag™ SDS-PAGE was performed with 5% polyacrylamide gels containing 50 μM Phos-tag™ acrylamide (Nard Institute, AAL-107) and 100 μM MnCl_2_ and according to manufacturer's instructions. After electrophoresis, Phos-tag™ acrylamide gels were transferred in SDS-containing transfer buffer (25 mM tris, 192 mM glycine, 20% (v/v) ethanol, 0.1% (w/v) SDS). Membranes were blocked in 5% (w/v) non-fat dried milk and incubated with the indicated primary antibodies, followed by incubation with LI-COR near infrared secondary antibodies. Immunodetection was carried out with a LI-COR Odyssey® CLx imaging system.

### FRET and FLIM Analysis

FLIM measurement of FRET was performed with a multiphoton microscope system as described previously (8, 23). A Nikon TE2000E inverted microscope, combined with an in-house scanner and Chameleon Ti:Sapphire ultrafast pulsed multiphoton laser (Coherent Inc.), was used for excitation of GFP (at 890 nm). Fluorescence lifetime imaging capability was provided by time-correlated, single-photon counting electronics (SPC 700; Becker & Hickl). A 40 × objective (NA 1.3) was used throughout (CFI60 Plan Fluor; Nikon), and data were acquired at 500 ± 20 nm through a bandpass filter (35–5040; Coherent Inc.). Acquisition times of ~300 s at low excitation power were used to achieve sufficient photon statistics for fitting, avoiding either pulse pile-up or significant photobleaching. Data were analyzed as previously described (65). The FRET efficiency is related to the molecular separation of donor and acceptor and the fluorescence lifetime of the interacting fraction by:

ηFRET = (R06/(R06 + r6)) = 1–τFRET τd,

where ηFRET is the FRET efficiency, R0 is the Förster radius, r is the molecular separation, τFRET is the lifetime of the interacting fraction and τd is the lifetime of the donor in the absence of an acceptor. The donor- only control is used as the reference against which all of other lifetimes are calculated in each experiment. τFRET and τd can also be taken to be the lifetime of the interacting fraction and non-interacting fraction, respectively. Quantification of FRET was made from all pixels within each cell that was analyzed. All image collection and data analysis were performed using TRI2 software (developed by Paul Barber, Gray Cancer Institute, London, UK).

### Surface Plasmon Resonance

Surface plasmon resonance measurements of L-selectin/FERM competition studies have been published previously (34). Human FERM domain of moesin (residues 1–297) was overexpressed and purified according to a previously published protocol (66). The expression plasmid encoding the open reading frame of human moesin FERM was a kind gift from A. Bretscher, Cornell, NY. To avoid covalent inactivation of essential side chains, the cytoplasmic tail of L-selectin was synthesized as a biotinylated peptide (conjugated to Arg-356), dissolved in 10 mM HEPES (pH 7.4), 150 mM NaCl, 0.005% (v/v) polysorbate 20 (HBS-P) and immobilized on streptavidin-coated sensor chip surface using a non-covalent sandwich system. Approximately 20 μM of the biotinylated L-selectin peptide was injected into the sensor chip, followed by a wash phase to remove excess unbound peptide. Next, 5 μM of the moesin FERM domain (dissolved in HBS-P) was injected into the flow cell at a flow rate of 5 mL/min. When the interaction readings stabilized, excess peptides were injected as outlined in Figure 4 and sensograms were produced to determine the extent of competition (deemed as a sharp drop in response units). Data were evaluated using the BIAevaluation software and regeneration of the sensor chip surface was achieved by injection of 100 mM NaOH followed by a wash phase in HBS-P and subsequent reloading of the sensor surface with 1 M biotinylated L-selectin cytoplasmic tail. All measurements were monitored at 25°C. All biotinylated/phosphorylated peptides were synthesized and purified by BrisSynBio at the University of Bristol UK.

### Molecular Dynamics

All Molecular Dynamics (MD) simulations presented in this work are based on the Martini force field (67, 68). Following the Martini philosophy, on average four heavy atoms plus associated hydrogens are grouped together into one interaction center, a so-called coarse-grained (CG) bead. Depending on the underlying chemical nature, a bead can be classified as polar, non-polar, apolar or charged, which determines the non-bonded interactions with other beads [see Marrink et al. (68) for details]. The Martini force field has been widely applied to study the interplay between lipids and proteins in a large variety of membrane environments [see many examples in Marrink et al. (69), Corradi et al. (70)].

The studied L-selectin models consist of a transmembrane (TM) and the cytoplasmic tail of 23 and 17 residues, respectively. The sequences of the human and mouse L-selectin tails are shown in Figure 4. Pymol (an open source graphics tool: http://www.ccp4.ac.uk/newsletters/newsletter40.pdf#page=44) was used to generate atomistic models of all L-selectin variants, which were subsequently transferred to CG level using the martinize script (71). In accordance with experimental data (72), the secondary structure of the TM domain was defined as an alpha-helix, whereas the tail was modeled as a random-coil.

For the lipid bilayers, two types of lipids were used: zwitterionic POPC (1-palmitoyl-2-oleoyl-sn-glycero-3-phosphatidylcholine, net charge q = 0e) and anionic PIP2 (1-palmitoyl-2-oleoyl-sn-glycero-3-phospho-(1-D-myo-inositol 4,5-bisphosphate), q = −4e). Parameters for POPC were obtained from Wassenaar et al. (73). A model for PIP2 has recently been parametrized (Sun) based on the PI (3, 4)P2 model (74). Here, we reduced the net charge of PIP2 to −4e as this is in a better agreement with experimental results (75). In this work, a POPC bilayer with 6% PIP2 lipids in the lower leaflet was studied. The simulation boxes were built using the tool insane (73), generating a lipid bilayer of 252 lipids per leaflet (12.1 ×12.1 ×14 nm^3^). L-selectin was inserted into the bilayer parallel to the membrane normal. All systems were solvated with standard CG water beads and neutralized with sodium counterions.

The simulations were performed with the software package Gromacs 2018.1 (76) thereby using simulation parameters in agreement with the “New-RF” parameters for Martini simulations (77): First, the systems underwent an energy minimization using the steepest-descent algorithm until the maximum force on any bead in the system did not exceed a value of 10 kJ mol−1 nm−1. After energy minimization the systems were equilibrated for 200 ns in an NVT ensemble with a reference temperature of 310 K, using a velocity rescaling thermostat (78), followed by an NPT equilibration for 400 ns using the Berendsen barostat (79) with a reference pressure if 1 bar, a time constant of 4 ps and an isothermal compressibility of 3 ×10^−4^ bar-1 that was coupled to the system in a semiisotropic way. During both equilibration procedures the backbone beads of L-selectin were restrained to their initial positions by a harmonic potential with a force constant of 1,000 kJ mol-1 nm-2. After equilibration, three independent productions runs, 5 μs each, were conducted without any positions restraints; here constant pressure was achieved by using the Parrinello-Rahman barostat (80, 81) with a time constant of 12 ps. For all simulations, periodic boundary conditions were applied and the integration time step was set to 20 fs. The first 1 μs of each production run was discarded, the remaining 4 μs were analyzed with the built-in analysis tools of Gromacs. The density profiles in Figures 5B,C, the data from each of the 3 production runs was pooled together, thus resulting in a total simulation time of 12 μs for analysis. For visualization purposes, the program VMD (82) was used.

### Parallel Plate Flow Chamber Assays

All flow experiments were performed using a 35 mm diameter Glycotech parallel plate flow chamber. Perfusion experiments were performed at 1.5 dyn/cm^2^ using a Harvard Apparatus 2000 PHD syringe pump. Perfusion media consisted of: RPMI supplemented with L-glutamine, 10% FCS, 1% penicillin/streptomycin, 50 μM β-mercaptoethanol, and 25 mM HEPES. Human Umbilical Vein Endothelial Cells (HUVEC–Lonza) were seeded onto 35 mm diameter glass coverslips (no. 1 thickness; VWR) that were pre-coated with 10 μg/mL fibronectin (37°C for at least 1 h). Before each perfusion assay, HUVEC were stimulated overnight (16 h) with 10 ng/mL carrier-free recombinant human TNF-α (R&D Systems). Each perfusion assay was performed by injecting a bolus of cells for 6 min, followed by just perfusion media (without cells) for the remaining 25 min. THP-1 cells were perfused at a density of 0.5 ×10^6^ cells per mL. THP-1 cells treated with 10 μM TAPI-0 required a preincubation time of 10 min at 37°C before perfusion over TNF-α-activated HUVEC. Note that 10 μM TAPI-0 was also supplemented in the perfusate. Stills were acquired once every 10 seconds using 10 × objective lens.

For FRET/FLIM analysis, coverslips were detached from the flow chamber after 15 min of flow, which is a period when protrusive activity is optimal, but ectodomain shedding is minimal (8, 23). Coverslips were immediately submerged in 4% (vol/vol) PFA solution (dissolved in PBS) for 10–15 min at room temperature. Cells were washed four to five times in PBS to remove excess PFA and permeabilized for 3 min in ice- cold PBS containing 0.1% (vol/vol) Nonidet P-40 substitute (Fluka). After gently washing off the permeabilization buffer, coverslips were treated with sodium borohydride as described in the method for AMC. After washing off the sodium borohydride, coverslips were blocked in 10% FCS containing FcR block (Miltenyi Biotec Ltd.) overnight at 4°C. Specimens were then labeled with Alexa Fluor® 633 phalloidin (Thermo Fisher). Coverslips were finally washed four to five times in PBS and mounted onto glass slides using fluorescence mounting medium (Dako).

## Data Availability

All datasets generated for this study are included in the manuscript/Supplementary Files.

## Author Contributions

AI conceived the project, performed experiments, and wrote the paper. AN performed flow experiments and analyzed data. KR performed flow experiments and analyzed data. MK, CS, and SM performed experiments and analyzed all the data involving Molecular Dynamics. JJ performed experiments, contributed to data analysis, and compiled artwork for manuscript. AR-G contributed to experimental design. JD performed experiments related to surface plasmon resonance. MP performed analysis of FRET/FLIM data.

### Conflict of Interest Statement

The authors declare that the research was conducted in the absence of any commercial or financial relationships that could be construed as a potential conflict of interest.

## Supplementary Material

Supplementary Video 1 Model of L-selectin interacting with the inner leaflet of the plasma membrane.

Supplementary Video 2 THP-1 cell generating a single protrusion during TEM.

Supplementary Video 3 THP-1 cell generating a two protrusions during TEM.

Supplementary Video 4 THP-1 cell generating a multiple protrusions during TEM.
